# Supplementary material for: Machine Learning Prediction of Cancer Cell Sensitivity to Drugs Based on Genomic and Chemical Properties
Source: PLoS One. 2013 Apr 30;8(4):e61318. doi: 10.1371/journal.pone.0061318 (PMC3640019; doi:10.1371/journal.pone.0061318)
Supplement: Text S1 — Performance measurements. (DOC) [file pone.0061318.s005.doc]

**Text S1: Measuring performance.**

The output of our method is the logarithm to base 10 of the IC_50_ in μM:

$$y= \log\left(IC_{50}\right)$$

$y$: Prediction of log(IC_50_) by our method

$IC_{50}$: Required concentration [μM] to eliminate 50% of the cancer cell population, computed as described in Garnett et al *Nature* 2012

We evaluated three different performance metrics, (i) one that captures the linear dependency of the prediction versus observation, (ii) a metric for outlining the variance of an assumed perfect prediction and (iii) additionally another metric that describes the average error of the model predictions:

(i) Pearson correlation coefficient (*R_p_*) describes the relationship of prediction and observation. *R_p_* is in a range from -1 to 1; negative correlations hint at inverse predictions (more predicted wrong than correct), *R_p_* of 0 correspond to a random relationship (no correlation), and positive correlations indicate linear behaviour with positive gradient:

$$R_{p}=\frac{\sum\overrightarrow{y}_{obs}\overrightarrow{y}_{pred}-\frac{\sum\overrightarrow{y}_{obs}\sum\overrightarrow{y}_{pred}}{n}}{\sqrt{\left(\sum\overrightarrow{y}_{obs}^{2}-\frac{1}{n}\sum\overrightarrow{y}_{obs}^{2}\right)\left(\sum\overrightarrow{y}_{pred}^{2}-\frac{1}{n}\sum\overrightarrow{y}_{pred}^{2}\right)}}$$

$n$: Size of the test set

$\overrightarrow{y}_{pred}$: Vector of observed/expected log(IC_50_) value

$\overrightarrow{y}_{obs}$: Vector of predicted log(IC_50_) value

(ii) Coefficient of determination (*R^2^*) measures the proportion of the variance of the data that is explained by the regression model. As regression model, we assume a linear function representing a perfect prediction, or put a differently, plotting a line through observation-by-observation points. The following definition of *R^2^* typically returns values in range from 0 to 1, where values closer to one indicate a good prediction and 0 suggest weak fitting of the observations. However, since the regression model is not data driven and rather a conservative assumption of being a perfect prediction, also negative values are possible in case the prediction is far off the observation.

$$R^{2}=1-\frac{\sum_{i=1}^{n} (y_{obs_{i}}-y_{pred_{i}})^{2}}{\sum_{i=1}^{n} (y_{obs_{i}}-\overline{y}_{obs})^{2}}$$

$n$: Size of the test set

$y_{obs}$: Observed/expected log(IC_50_) value

$\overline{y}_{obs}$: Average of all observed log(IC_50_) values

$y_{pred}$: Predicted log(IC_50_) value

(ii) Root mean square error (*RMSE)* provides an average of the error across all predictions made by the models:

$$RMSE=\sqrt{\frac{1}{n}\sum_{i=1}^{n} (y_{obs_{i}}-y_{pred_{i}})^{2}}$$

$n$: Size of the test set

$y_{obs}$: Observed/expected log(IC_50_) value

$y_{pred}$: Predicted log(IC_50_) value
