# Supplementary material for: Machine Learning Prediction of Cancer Cell Sensitivity to Drugs Based on Genomic and Chemical Properties
Source: PLoS One. 2013 Apr 30;8(4):e61318. doi: 10.1371/journal.pone.0061318 (PMC3640019; doi:10.1371/journal.pone.0061318)
Supplement: Text S2 — Comparison of imputation methods and machine learning approach. (DOC) [file pone.0061318.s006.doc]

**Text S2: Comparing performance of imputation methods and machine learning approach.** A straightforward way to estimate missing IC_50_ values is to impute them from the rest of the values. We therefore compared our feature-based approach against conventional imputation methods on our blind test. We used well-known reference methods from the R package “imputation” v1.3 developed by Jeffrey Wong: Singular Value Threshold (SVT), Singular Value Decomposition (SVD), and k-Nearest Neighbor (kNN) imputation. To estimate the parameters of each imputation method, we used cross validation as suggested in the example code of the R package. SVT imputation obtained 0.06 *R_p_* and 1.66 *RMSE*. The SVD imputation improved the performance to 0.37 *R_p_* and 1.54 *RMSE*. The best performing imputation method is kNN with 0.71 and 1.10 *R_p_* and *RMSE*, respectively. Our method (in this case a neural network) including genomic and chemical drug properties outperformed all imputation methods with an *R_p_* of 0.79, R^2^ of 0.72 and *RMSE* of 0.97. It is important to note that imputation techniques, unlike feature-based machine learning, cannot be used to extrapolate, i.e. to predict IC50s for unseen cell lines or drugs.
