# Supplementary material for: Machine Learning Prediction of Cancer Cell Sensitivity to Drugs Based on Genomic and Chemical Properties
Source: PLoS One. 2013 Apr 30;8(4):e61318. doi: 10.1371/journal.pone.0061318 (PMC3640019; doi:10.1371/journal.pone.0061318)
Supplement: Text S3 — Random Forest. (DOC) [file pone.0061318.s007.doc]

**Text S3: Random Forest.** In model building, we used the same training set, features and performance measures as in the neural network model. However, unlike in the neural network and for the sake of efficiency, we did not tune any parameter of the Random Forest and hence the 8-fold cross-validation was slightly different (i.e. the same test set used by the neural network model, but exploiting more training data with a control parameter-free Random Forest). Here all seven partitions are exclusively used for training and the last partition for test. As usual, the prediction on the eight independent folds, one per each regression, is averaged.

In the case of randomly constructed partitions, *RMSE* was 0.84, R^2^ was 0.72 and the *R_p_* was 0.85. In the case of leaving cells out partitions, *RMSE* was 0.85, R^2^ was 0.71 and the *R_p_* was 0.84. The performance on the blind test was *RMSE* was 1.00, R^2^ was 0.59 and the *R_p_* was 0.78. Overall, these results are very similar to those obtained with the neural network model. Each machine learning model was independently trained and validated using different computer codes, which further supports the robustness of our results.
